# A Genome-Wide Tethering Screen Reveals Novel Potential Post-Transcriptional Regulators in *Trypanosoma brucei*

**DOI:** 10.1371/journal.ppat.1004178

**Published:** 2014-06-12

**Authors:** Esteban D. Erben, Abeer Fadda, Smiths Lueong, Jörg D. Hoheisel, Christine Clayton

**Affiliations:** 1 Zentrum für Molekulare Biologie der Universität Heidelberg (ZMBH), DKFZ-ZMBH Alliance, Heidelberg, Germany; 2 Division of Functional Genome Analysis, Deutsche Krebsforschungszentrum (DKFZ), Heidelberg, Germany; Yale School of Public Health, United States of America

## Abstract

In trypanosomatids, gene expression is regulated mainly by post-transcriptional mechanisms, which affect mRNA processing, translation and degradation. Currently, our understanding of factors that regulate either mRNA stability or translation is rather limited. We know that often, the regulators are proteins that bind to the 3′-untranslated region; they presumably interact with ribonucleases and translation factors. However, very few such proteins have been characterized in any detail. Here we describe a genome-wide screen to find proteins implicated in post-transcriptional regulation in *Trypanosoma brucei*. We made a library of random genomic fragments in a plasmid that was designed for expression of proteins fused to an RNA-binding domain, the lambda-N peptide. This was transfected into cells expressing mRNAs encoding a positive or negative selectable marker, and bearing the “boxB” lambda-N recognition element in the 3′-untranslated region. The screen identified about 300 proteins that could be implicated in post-transcriptional mRNA regulation. These included known regulators, degradative enzymes and translation factors, many canonical RNA-binding proteins, and proteins that act via multi-protein complexes. However there were also nearly 150 potential regulators with no previously annotated function, or functions unrelated to mRNA metabolism. Almost 50 novel regulators were shown to bind RNA using a targeted proteome array. The screen also provided fine structure mapping of the hit candidates' functional domains. Our findings not only confirm the key role that RNA-binding proteins play in the regulation of gene expression in trypanosomatids, but also suggest new roles for previously uncharacterized proteins.

## Author Summary

Survival and adaptation of trypanosomatids to new surroundings requires activation of specific gene networks. This is mainly achieved by post-transcriptional mechanisms, and proteins that bind to specific mRNAs, and influence degradation or translation, are known to be important. However, only few such proteins have been characterized to date. The trypanosome genome encodes over 150 proteins with conserved RNA-binding domains, and it is very likely that additional proteins that do not have such domains could also modulate mRNA fate. Here, we report the results of a genome-wide screen to identify mRNA-fate regulators in *Trypanosoma brucei*. We used a method called “tethering” to artificially attach protein fragments to an mRNA. Our findings confirmed the role of RNA-binding proteins in the regulation of mRNA fate, and also suggested such roles for many other proteins, including some metabolic enzymes. Our results should serve as a useful resource. Moreover, the tethering screen approach could readily be adapted for use in other organisms.

## Introduction

Kinetoplastid protists are exposed to environmental challenges in the host and vector, necessitating extensive changes in gene expression. In kinetoplastids, most protein-coding genes are transcribed by RNA polymerase II in unidirectional clusters. Individual mRNAs are excised by 5′ *trans* splicing, with addition of a 39mer spliced leader RNA, and by 3′ polyadenylation [1]. At the level of individual pol II-transcribed open reading frames (ORFs), there is no evidence for control of expression via regulated transcription initiation. Trypanosomes and related parasites are therefore mainly dependent on post-transcriptional mechanisms for the control of gene expression [2], and thus for both survival and pathogenesis.

Control of gene expression at the post-transcriptional level is essential in all organisms, and RNA-binding proteins (RBPs) play critical roles at all stages: RNA processing, transport, stability and translation. The interactions of RBPs with cytosolic mRNAs - often, but not always, with the 3′-untranslated region (3′-UTR) - can affect transcript stability, translational efficiency, or both [3]. In this way, RBPs can modify and regulate every step of RNA metabolism and function. Recent studies in different organisms have revealed that the repertoire of RBPs is far greater than anticipated [4]–[7]. Although members of all classical RBP domain families were abundantly represented in these analyses, several novel and unexpected candidates with no RNA-related ontology or domain homology were seen to interact with RNA. In addition to novel proteins containing low complexity amino acid repeat motifs, metabolic enzymes, and enzymes with potential RNA- and protein-modifying activities were found. However, for most of these novel RBPs, the functional consequences of the mRNA binding remain unknown.

In trypanosomatids, RBPs are key factors in gene expression regulation. Over a hundred RBPs have been predicted to exist in trypanosomes, based on their containing canonical RNA-binding domains: RNA recognition motif (RRM), CCCH zinc fingers, and pumilio or PUF domains. Some of these are already known to be involved in the regulation of differentiation, development, the cell cycle and rRNA processing (recently reviewed in [8]). Most of the characterized RBPs control mRNA abundance by stabilizing the target transcript (for example *Tb*ZPF3 [9], *Tb*ZC3H11 [10], *Tb*ZC3H20 [11], *Tb*PUF9 [12]) or by increasing translation (ALBA domain proteins, [13]). In mammalian cells, several proteins (such as BRF1, TTP, AUF1 and TIA-1) have been shown actively to destabilize mRNAs or to act as translation silencers ([14]. In trypanosomatids, although indirect results suggest that some proteins cause degradation of target transcripts this has never been shown directly (examples are UBP1 [15], PUF6 [16] and PUF5 [17].) There is evidence that one protein, RBP10, can suppress translation when attached to an mRNA [18]. With the single exception of ZC3H11 [19], the mechanisms by which trypanosome proteins determine mRNA fate are not known.

The functions of RBPs *in vivo* can be studied by “tethering” them to a reporter mRNA. In this technique, the protein under study is attached to the UTR of an mRNA reporter through an artificial RNA-protein interaction [20]. Hence, the functional activity of a protein can be studied independent of its intrinsic ability to bind to RNA. This assay has been applied successfully for numerous proteins in diverse organisms [21] and has proven useful in the analyses of the precise function of essential genes [22], mapping of protein function [10], dissecting functional maps of protein complexes [23] and visualizing tagged mRNAs [24]. The bacteriophage lambda-N protein is often used in the tethered function assay [25]. N-protein regulates bacterial transcriptional anti-termination by binding to a 15-nucleotide RNA hairpin, called boxB, within nascent transcripts [26]. The N-peptide is used in tethering assays because its small size (only 22 amino acids) limits the extent of likely effects on the function of the fused protein, and because of its high affinity interaction with boxB RNA.

In this paper, we use the “tethering” approach in a genome-wide screen for post-transcriptional regulators in *Trypanosoma brucei*. We have been able to take advantage of the excellent tools for advanced genetics and very high efficiency transfection of trypanosomes [27]. Also, since introns are almost completely absent and coding sequences are closely spaced, all open reading frames could be represented in a library made from genomic DNA. We were able to map functional domains on well-characterized proteins and found evidence for functions of many RBPs. RNA-binding properties were also analysed using a targeted protein array. The results greatly increase our knowledge of the possible regulatory repertoire in trypanosomes and provide functional annotation for many previously uncharacterized genes.

## Results

### Regulation of selectable markers by tethered proteins

**Figure 1:**
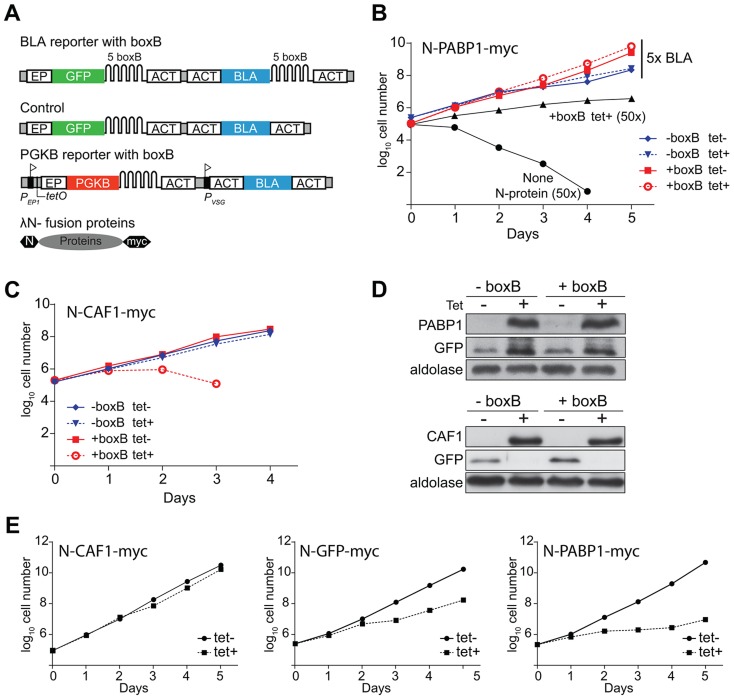
Proof of concept of the tethering approach. (**A**) Schematic diagram of reporter mRNAs and tethered proteins, not to scale. Above: *BLA* and *GFP* reporter mRNA with five boxB recognition sites in the 3′-UTR. Middle: Control *BLA* 3′-UTR mRNA without boxB. Below: PGKB mRNA with boxB recognition sites at the 3′-UTR. A tetracycline-inducible EP1 procyclin promoter (flag) drives *PGKB* expression. The downstream promoter, P_VSG_, gives constitutive expression of the resistance marker. Bottom: Fusion protein with N-terminal lambda-N peptide and C-terminal myc tag. (**B**) Cumulative growth curves of cells expressing the *BLA* reporters with tetracycline-inducible lambda-N-PABP1, in the presence of 5x blasticidin (25 µg/ml) with or without tetracycline. For comparison, cells expressing lambda-N-PABP1 and non-transfected cells (without N-protein, parental cell line) grown in 50x blasticidin (250 µg/ml) are shown. The +boxB cells with lambda-N-PABP express a little of the lambda-N fusion even in the absence of tetracycline (panel D), which may result in slightly better growth in the presence of 25 µg/ml blasticidin. (**C**) Cumulative growth of cells containing the *BLA-boxB* reporter expressing N-CAF1-myc in the presence or absence of tetracycline and 5x blasticidin. (**D**) Western blot analysis of N-PABP1-myc, N-CAF1-myc and N-GFP proteins. Expression of PABP1-myc and CAF1-myc was detected with anti-myc, using aldolase as loading control. (**E**) Effect of tethered PABP1, GFP and CAF1 on growth after tetracycline-mediated induction of both PGKB and tethered protein expression.

To screen for proteins that increase gene expression, we used a reporter cell line constitutively expressing the blasticidin resistance (BLA) protein. The *BLA* mRNA contains five copies of the *boxB* hairpin RNA sequence between the resistance cassette and the actin (*ACT*) 3′-UTR (*BLA-B-ACT*). As a secondary reporter, a cassette encoding *GFP* (*GFP-B-ACT*) was introduced upstream of the blasticidin reporter (Figure 1A). Cell lines containing the *BLA* reporter mRNA lacking the *boxB* element were used as controls. The tandemly arranged reporters were integrated into the trypanosome alpha-beta tubulin repeat, and should be transcribed constitutively by RNA polymerase II reading through from upstream.

We first introduced a plasmid that inducibly expressed trypanosome poly(A)-binding protein 1 (PABP1) with the lambda-N peptide at the N-terminus and a *myc* tag at the C-terminus (N-PABP1-myc). Tethering of N-PABP1 to a trypanosome mRNA is known to increase mRNA stability and reporter expression [28]. As we had hoped, co-expression of N-PABP1-myc enabled the cells to grow at 250 µg/ml blasticidin - 50 times the normal concentration - whilst cells expressing only the *BLA-B-ACT* reporter were rapidly killed (Figure 1B). At 25 µg/ml blasticidin (5-fold normal), cells containing the *BLA* reporter with the *boxB* element grew slightly faster than the control (Figure 1B); this was independent of tetracycline addition, probably because a small amount of N-PABP1-myc was expressed in the absence of tetracycline (Figure 1D). As expected, tethering of PABP1-myc also increased GFP expression (Figure 1D). To find out whether we could also detect the activity of proteins that decreased expression, we tethered the deadenylase CAF1, which is known to result in reporter mRNA degradation [29]. Indeed, expression of the N-CAF1-myc decreased cell survival under low (25 µg/ml) blasticidin pressure (Figure 1C). Inhibition of GFP protein expression in both cell lines confirmed specificity (Figure 1D).

We next designed a screen that would positively select for proteins that decreased expression upon tethering. In bloodstream trypanosomes, inducible expression of cytosolic phosphoglycerate kinase B (*PGKB*) is lethal [30]. We therefore created a cell line with a tetracycline inducible *PGKB* open reading frame followed by 5 *boxB* copies at the 3′-UTR (Figure 1A). Upon tetracycline induction, the parasites died (not shown). As expected, co-expression of N-CAF1-myc (“destabilizing”, Figure 1E) expression conferred a selective advantage while N-GFP-myc (“neutral”, Figure 1F) and N-PABP-myc (“stabilizing”, Figure 1G) did not rescue the parasites. We concluded that our two complementary selections could be used to discover proteins affecting mRNA-fate.

### Screening for modulators of trypanosome mRNA-fate

To screen for proteins involved in mRNA-fate regulation, a plasmid library was constructed using randomly sheared trypanosome genomic DNA. The plasmid backbone was designed such that transcription of each genomic DNA fragment could be induced with tetracycline, and the resulting proteins would contain the lambda-N peptide at the N-terminus. 3×10^6^ independent clones were obtained with an average insert size (as judged by plasmid-specific PCR) of about 1.2 kbp. Based on the gene density within the coding regions of trypanosome chromosomes we expected approximately one in twelve plasmids to encode an authentic trypanosome protein fragment fused in frame to the N-peptide. This would indicate that our plasmid pool represents a ∼10-fold coverage of the open reading frames.

The inducible library was transfected into bloodstream cells expressing both the *BLA-B-ACT* and *GFP-B-ACT* reporters, using site-specific endonuclease-enhanced transfection [31]. In the first experiment (BLA-A), 1.8 million clones were obtained and in a second (BLA-B), 3.3 million clones (Supplementary Figure S1). To determine the sequence coverage of the fusions in the trypanosome populations, the plasmid inserts were amplified by PCR, using primers located within the lambda-N peptide and immediately 3′ to the cloning site. Control experiments with the resistance mRNA lacking the *boxB* elements were performed once with 3.1 million clones (experiment BLA-C).

**Figure 2:**
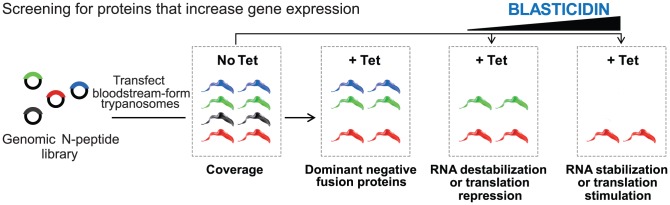
Schematic of the overexpression library and the growth conditions analyzed. After transfection, the library was grown under non-inducing (tet −) and inducing conditions (tet +) with increasing concentration of blasticidin and then, genomic DNA was isolated from survival populations. Adaptor-ligated sequencing libraries were prepared from specific PCR reactions and Illumina sequenced. Illumina sequencing reads containing a backbone vector junction sequence were then mapped to the *T. brucei* reference.

We induced N-peptide fusion protein expression for 24 h and then grew cells for four days under different conditions. Clones that were unable to grow after tetracycline induction must express proteins that have intrinsic dominant-negative effects, unrelated to blasticidin resistance (Figure 2, black cloned inserts and trypanosomes). In addition, after 24 h induction we grew the cells for four days in various concentrations of blasticidin. To detect fusion proteins that decrease mRNA stability or translation (Figure 2, blue) we grew the cells in 1x (experiment BLA-A) or 2x (experiment BLA-B) levels of blasticidin. No significant effect on overall population growth was seen (Supplementary Figure 1A, C). To detect fusion proteins that increase mRNA stability or translation (Figure 2, red) we grew the cells in 6x (experiment BLA-A) or 10x or 20x (experiment BLA-B) levels of blasticidin. In this case, growth was impaired but then the cells started to recover, indicating rescue by lambda-N fusions that could enhance *BLA* expression (Supplementary Figure 1A, C). In parallel, we run a similar experiment with the cells carrying the *BLA* reporter without *boxB* elements (Supplementary Figure 1D, experiment BLA-C). The cloned genomic DNA fragments were in each case recovered by plasmid-specific PCR. Examination of small aliquots from the reactions revealed similar-looking smears of many products for all populations except the high blasticidin treated cells (Supplementary Figure 1B, E). All PCR mixtures were subjected to high-throughput sequencing.

To screen for protein fragments that impaired expression, the plasmid library was transfected into bloodstream-form cells expressing the inducible *PGKB-B-ACT* reporter (Supplementary Figure 2A). Three independent experiments were done, resulting in 0.65 million (Library PGKB-A), 1.2 million (Library PGKB-B) and 4.8 (Library PGKB-C) million independent clones. Expression of both *PGKB* and the lambda-N fusions was induced with tetracycline and cells were grown in the presence of inducer for 5 days. In the libraries, the induced PGKB only slightly affected growth. Presumably, some of the parasites had lost inducible expression of the lethal protein during the transfection procedures. This is a common problem in trypanosomes that inducibly express toxic RNAs or proteins. Nevertheless, expression of lambda-N fusions did confer a modest growth advantage (Supplementary Figure 2B). Again, the selected plasmid inserts were amplified (Supplementary Figure 2C) and sequenced.

### The screen detects known expression activators

**Figure 3:**
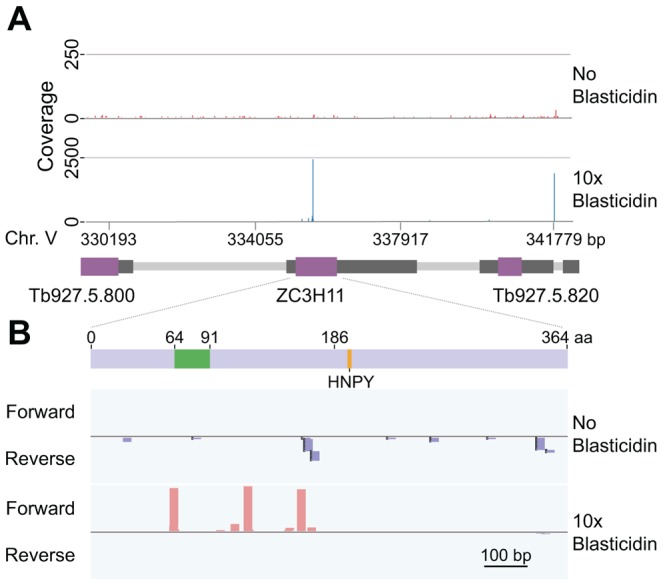
Results for ZC3H11. (**A**) Sequence reads that were linked to the lambda-N peptide were extracted, mapped onto the trypanosome genome and visualised using the Integrative Genomics Viewer (IGV) tool. The Figure shows the sequence coverage plots for a ∼12 kbp region of chromosome 5. Open reading frames are in purple and mRNA untranslated regions in dark grey. Reads were derived from tetracycline-induced cells grown without blasticidin (upper panel), or with the 10x blasticidin concentration (bottom panel). For better visualization, the plotted Y scales display different range values. In the 10x blasticidin plot, note the cluster over ZC3H11; the isolated “pile” downstream of Tb927.5.820 would be removed later in the filtering process. (**B**) These are the same results as in (A), but in close-up focusing on the ZC3H11 (Tb927.5.810) open reading frame. Each “stack” represents a unique target fragment, mapped to forward (plus) and reverse (minus) strands, for induced cells grown without (upper panel) and with (bottom panel) blasticidin pressure. Light blue boxes indicate the ZC3H11 coding sequence (transcribed left to right) while zinc finger domain and HNPY motif (residues 186–189) are indicated in green and orange respectively. The minimum region that was required for activity in tethering assays was the C-terminal part, starting at amino acid 186.

Sequencing reads from the blasticidin expression experiment were mapped to the *T. brucei* reference after trimming of the non-trypanosome boundary sequences. Only reads that were in frame with the lambda-N peptide were considered. Results are tabulated in Supplementary Tables S1, S2, S3, with Supplementary Table S1 showing the reads for all mapped in-frame locations at the nucleotide level. Before selection, reads were fairly uniformly distributed with no strand or coding-region preference. However, under inducing conditions, in-frame coding-region reads on particular genes were strongly enriched. Figure 3A shows an example of aligned reads from the second blasticidin selection experiment, in the ∼12 kb region that surrounds the gene encoding the zinc finger protein ZC3H11. After selection, the accumulation of reads over *ZC3H11* was striking. Overall there were about 50-fold more in-frame reads from the *ZC3H11* open reading frame after selection than in the unselected population.

We preciously characterized the activity of ZC3H11 using the tethering assay, with a chloramphenicol acetyltransferase (*CAT*) reporter mRNA. We found that the entire region downstream of residue 185 was required for activity [19]. This portion contains, at residues 196–199, a four-residue motif (HNPY) that is required for interaction with the post-transcriptional regulator MKT1, and beyond that an additional region that interacts with the PABP-interacting protein PBP1 [19]. Consequently, any lambda-N fusion that commenced beyond residue 185 should not be selected, and fusions that commenced earlier, but did not include the C-terminus, should also be excluded. Figure 3B, which shows the individual reads, illustrates this: without selection reads were scattered on forward and reverse strands, but after selection, only fusions that commenced upstream of and including nt 477 (residue 159) showed enrichment. However, detailed analysis showed that a fusion that should have been selected, commencing at residue 167, was not enriched. This particular fusion protein may have non-functional folding pattern, or it may be truncated at the C-terminus. Interestingly, overall, many fusions that were selected by blasticidin were not detected in the population prior to selection.

To further check the results we examined additional genes whose activities had previously been studied in the tethering assay. Both PABP1 and PABP2 yielded multiple clones that increased blasticidin resistance. Averaging the entire open reading frames, PABP1 gave 80-fold more reads after 6-fold or 10-fold blasticidin selection than were present without blasticidin, and PABP2 gave 25-fold read-count enrichment. Pab1p-binding protein, PBP1 - which is also active in the tethering assay [19] - gave over 150-fold enrichment. In each case, it is possible to deduce approximately, from the positions of selected fusions, the portion of the protein that is required for activity in the tethering assay.

### Activators include many proteins with RNA-binding domains

We now analyzed the entire dataset from the blasticidin selection experiment (Supplementary Table S1). To do this, we used a list of 6933 non-redundant protein-coding sequences predicted in the assembled *T. brucei* genome ([32] with modifications) (Supplementary Table S2). We obtained at least one in-frame read for more than 6700 (>96%) of these. The mapped sequence reads represented over 340 thousand independent locations, equivalent to >10 locations and >50 reads for each CDS (Supplementary Table S2). For data analysis we included reads that started from position −36 relative to the ATG, to maximize retention of N-terminal sequences. We also excluded all reads that would include less than 6 amino acids of the protein C-terminus.

To obtain an overview of the data, and a list of candidate activators, we selected proteins from which at least two different fragments showed read-count enrichment of at least 3-fold, and there was also at least 3-fold read-count enrichment when the reads for the whole ORF were considered (see Methods for details). We will henceforth discuss these proteins as activators, although strictly speaking we have only shown activation activity for at least two fragments. If only one or two clones from an ORF contain the entire region required for activity, averaging over the entire ORF will eliminate real effects. Our selection was therefore biased against long proteins, and against any proteins for which regions near the N-terminus are required for activity. A few proteins that were not covered at all in experiment BLA-A were also excluded. For these reasons it is essential to refer to Supplementary Table S1 in individual cases.

**Figure 4:**
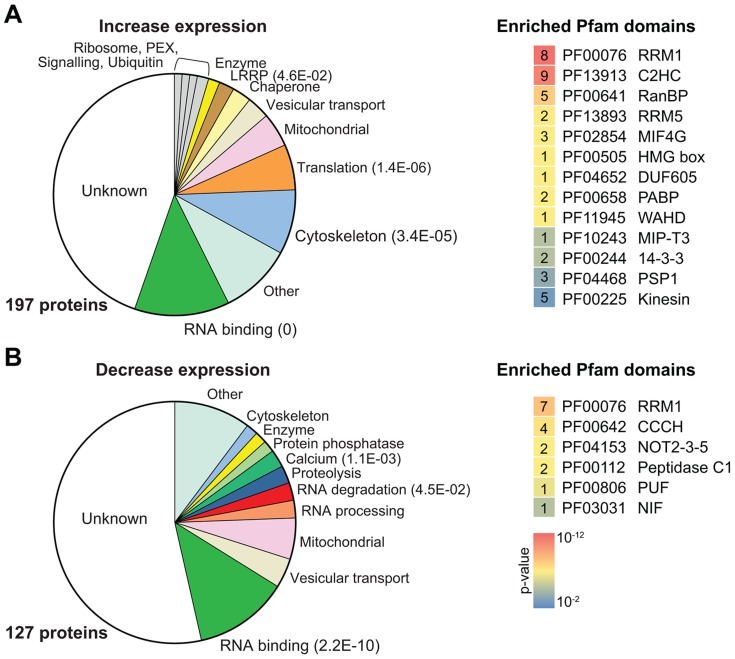
Enriched protein categories and Pfam domains. (**A**) Proteins for which fragments increased BLA resistance are classified by category. Different classes of gene function are shown in different colors. For highly enriched groups (Fisher's exact test, P<0.05) the E-values are shown in parentheses. On the right, all Pfam domains that were significantly enriched are shown (P<0.01, Fisher's exact test); lower p-values are represented in red, higher p-values are represented in blue. The numbers of each domain identified are shown in the corresponding boxed. (**B**) Proteins for which fragments suppressed PGKB expression. Details are as in (A).

Applying the stringent criteria listed above, there were 197 putative up-regulating proteins. We classified them functionally using TritrypDB annotations supplemented by other published information (Supplementary Table S3). Although a huge variety of proteins was classified among the activators (Figure 4A), known activators were included and both RBPs and translation factors were strongly enriched (Figure 4A, Table 1). We also found unexpected enrichment of cytoskeleton-associated proteins. Although a role for the cytoskeleton as translation regulator is possible [33], more probably the enrichment is an artifact of the alignments because the proteins contain sequence repeats (discussed in more detail later).

**Table 1:** RNA-binding proteins and translation factors that increase BLA resistance.

Locus number	Description	Number locations	Fold per CDS
RNA binding proteins			
Tb927.4.400	DRBD7	3	11.5
Tb927.10.11270	RBP23	7	23.6
Tb927.7.880	RBP25	5	8.7
Tb927.10.13720	RBP29	8	10.1
Tb927.6.4440	RBP42	15	10.6
Tb927.9.8740	DRBD3 (PTB1)	2	14.6
Tb927.11.14100	DRBD4 (PTB2)	10	11.1
Tb927.3.3960	DRBD6A	7	42.8
Tb927.3.790	ZC3H6	10	4.8
Tb927.5.810	ZC3H11	10	24.0
Tb927.7.2660	ZC3H20	9	122.1
Tb927.7.2670	ZC3H21	7	65.1
Tb927.8.4020	ZC3H24	6	26.2
Tb927.8.4070	ZC3H25	7	26.2
Tb927.8.4120	ZC3H26	7	26.2
Tb927.9.9450	ZC3H28	24	25.3
Tb927.10.5150	ZC3H31	4	4.2
Tb927.10.12330	ZC3H34	7	20.9
Tb927.10.12760	ZC3H36	7	50.0
Tb927.10.12780	ZC3H37	10	64.7
Tb927.10.12800	ZC3H38	15	48.6
Tb927.10.14930	ZC3H39	6	6.8
Tb927.10.14950	ZC3H40	16	15.4
Tb927.2.4710	TRRM1	5	6.0
Tb927.9.9290	PABP1	11	58.4
Tb927.9.10770	PABP2	10	45.7
Tb927.11.6240	2 weak RBDs	10	14.1
Tb927.3.1910	histone RBD	4	6.2
Other known factor			
Tb927.8.4540	PBP1	15	91.6
Translation factors			
Tb927.7.1710	DOM34	2	14.3
Tb927.3.2900	EF-2 alpha	6	6.7
Tb927.11.11770	eIF4E3	5	88.6
Tb927.6.1870	eIF4E4	18	23.3
Tb927.9.5460	eIF4G2	10	5.1
Tb927.11.10560	eIF4G4	8	38.5
Tb927.8.4500	eIF4G5	9	26.5
Tb927.11.5840	SUI1 homologue	2	20.6
Tb927.11.12670	TYW3 homologue	4	14.2

The number of locations is the number of positions at which a fusion protein caused at least a 3-fold increase in reads per million in the BLA6x or BLA10x condition. ‘Fold per CDS’ is the average increase for the whole CDS, including experiment A (BLA6x, 30 µg/ml) and experiment B (BLA10x, 50 µg/ml). Other proteins that are thought to increase stability and/or translation were positive in only one experiment or with only one fragment. These included the pumilio domain protein PUF9 [12], the ALBA proteins [13], the small CCCH protein ZFP3 [9], [58], [59] the 33 kDa subunit homolog of the cycle sequence binding protein (CSBP, [60] and RBP33 [34].

Proteins containing Pfam domains related to RNA binding (for example, RRM and zinc finger variants) were the most significantly increased group (Figure 4A). This is very encouraging since these proteins might be expected to have activity even when not tethered via lambda-N (Table 1). The 25 active proteins include 15 zinc finger proteins and 10 RRM domain proteins. Several of the RBPs that increased expression in our tethering screen have been shown to increase the abundance or translation of their target mRNAs, but have not previously been analyzed by tethering (Supplementary Figure S3). These include ZC3H20 [11], PTB1/DRBD3 [34], [35], and PTB2/DRBD4 [35]. RBP42, which was also found in the enhancing group, is associated with coding and non-coding regions of abundant mRNAs [36], which would be consistent with a stabilizing function.

### Translation initiation factors tethered to the 3′-UTR can increase gene expression

Proteins involved in translation - especially initiation factors - were over-represented in the activating population. The trypanosome genome encodes multiple homologues for the eIF4A (two), eIF4E (four) and eIF4G (five) subunits. Of these, only eIF4E3 and eIF4E4, which are in complex with eIF4G4 and eIF4G3 respectively, are thought to be active in *T. brucei* translation initiation [37]. Supporting this, we found different fragments of the initiation factors eIF4E3 and 4 to increase reporter expression; but the full set of eIF4Gs appeared to be active (Supplementary Figure S4). Other high-scoring proteins related to translation are listed in Table 1.

### Proteins that decrease gene expression: XRNA and the NOT complex

We now examined the fusion protein fragments that were positively selected in the presence of *PGKB-B-ACT* (for details, see Methods). The strongest suppressors of expression were also negatively selected in the BLA experiments after addition of 5–10 µg/ml (1–2x) blasticidin (Supplementary Tables S4 and S5). We found 127 proteins that gave reproducible repression of gene expression (Supplementary Table S3, and S4 sheets 3 & 4; for definition of “reproducible” see methods). As for the activators, the discussion below refers to whole proteins although we have actually shown activity only for at least two fragments. Reassuringly, three components of the CAF1/NOT deadenylation complex NOT2, NOT11 (C2ORF29) and NOT5 [38] reproducibly suppressed *PGKB* expression, suggesting these subunits could recruit active NOT/CAF1 complex to associated mRNA. In contrast, neither CAF1 nor UBP1, both of which are active as full-length proteins (this paper, [28], [29]) were selected, and neither were the two CAF/NOT complex proteins CNOT10 or CAF40, presumably because larger fragments or the intact protein are required. The ORF that encodes the 5′-3′ exoribonuclease XRNA [39] is over 4 kb long and three tethered fragments that gave really clear suppression all contained the C-terminal quarter of the protein (Supplementary Figure S5). As shown for both human and *Drosophila melanogaster* XRN1 [40], we speculate that the C-terminus may recruit other proteins that initiate mRNA degradation.

### Repression by RNA-binding proteins and 4E-IP

Multiple potential RBPs were able to suppress *PGKB* expression. These included RBP10, which was previously shown to inhibits translation if tethered to a reporter [18] and DRBD12 (Tb927.7.5380), which was shown to destabilize ARE-containing targets [41] (Supplementary Figure S5). In total, we found 16 RBPs conferring a clear negative effect on reporter expression, from which 7 were zinc finger proteins, 8 had a RRM motif and one, PUF3, a pumilio domain (Table 2). In mammalian cells, cytosolic pumilio domain proteins are mostly known as translational repressors [42].

**Table 2:** Selected factors that suppress PGKB expression.

Locus number	Description	Number locations	Number experiments	PGKB mean	BLA mean
RNA binding proteins					
Tb927.11.12120	RBP9	5	4	10.52	0.05
Tb927.8.2780	RBP10	4	2	2.63	0.94
Tb927.11.5850	RBP38	7	3	2.89	0.51
Tb927.6.3480	DRBD5	5	3	2.47	0.65
Tb927.4.400	DRBD7	13	4	3.39	0.85
Tb927.3.3940	DRBD11	14	2	2.96	0.77
Tb927.8.710	DRBD17	17	3	1.86	0.48
Tb927.10.310	PUF3	9	2	2.85	1.85
Tb927.3.5250	ZC3H8	16	4	2.95	0.48
Tb927.5.1580	ZC3H13	12	4	3.17	0.18
Tb927.6.4720	ZC3H15	7	5	4.40	0.05
Tb927.7.2680	ZC3H22	8	3	2.74	0.64
Tb927.9.9520	ZC3H29	4	4	2.59	0.05
Tb927.10.12740	ZC3H35	9	4	2.33	0.17
Tb927.7.5380	dsRNA-BD	10	3	2.13	0.55
RNA degradation					
Tb927.8.1960	NOT11	7	3	2.37	0.52
Tb927.6.850	NOT2	8	3	2.96	1.66
Tb927.3.1920	NOT5	6	3	2.35	0.75
Tb927.11.15000	SMN-like	4	4	2.40	0.53
Translation factor					
Tb927.9.11050	4E-IP	17	5	4.49	0.01

The number of locations is the number of positions with at least 3-fold enrichment in the PGKB experiments; Number of experiments: this is the number of experiments (out of 3 PGKB and two BLA) that showed either a 2-fold enrichment per CDS (PGKB) or a 2-fold depletion per CDS (for BLA). PGKB mean: arithmetic mean of enrichment factors for all 3 PGKB experiments. BLA mean: arithmetic mean of enrichment factors for the 2 BLA experiments.

One of the most dramatic and reproducible suppressors we found was the eIF4E-interacting protein. The *Leishmania* homologue of this protein was recently shown to interact with eIF4E1 in a stage-specific fashion [43], and it was suggested that this keeps eIF4E1 inaccessible for translation. Finally, several proteases, peptidases and components of the ubiquitination machinery were found to suppress expression when tethered. We speculate that in these cases the nascent polypeptide was subject to proteolysis.

### Identification and validation of novel post-transcriptional regulators

For many of the “hits” in our experiments, there was no previous indication for involvement in post-transcriptional regulation. 68 proteins that decreased expression, and 88 that increased it, have no previously annotated function at all (Figure 4B). There are also some metabolic enzymes in the list. It is tempting to speculate that these have regulatory function in addition to catalytic activity, as suggested for animal cells [44], [45]. Mass spectrometry studies of proteins poly(A)+ RNA in human cells catalogued more than 40 metabolic enzymes [4], [5].

**Figure 5:**
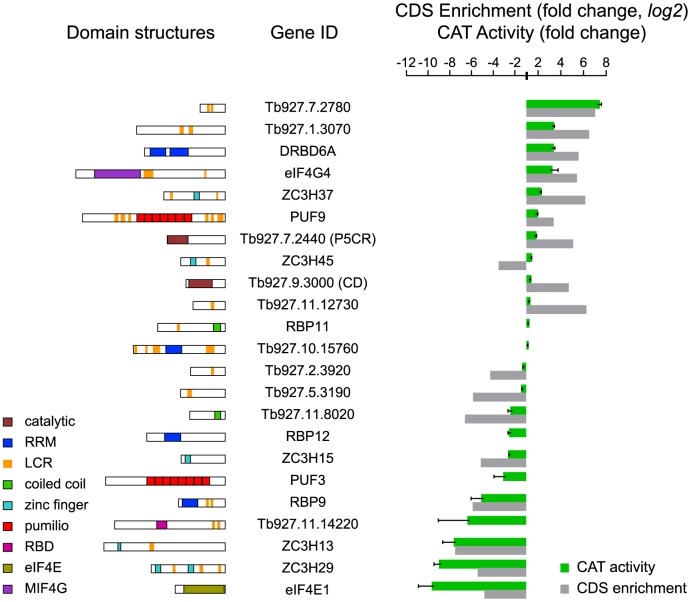
Validation of mRNA-fate regulators. Expression of CAT in cells expressing different myc-tagged lambda-N-fusion proteins was assayed after 24 h tetracycline induction and activities were expressed relative to a control with no lambda-N protein (*green*). Results are arithmetic mean ± standard deviation of at least 3 independent clones. As comparison, the fold per CDS (log2 values) are shown (*grey*). (*left*) Domain structures of analysed *T. brucei* proteins as detected by SMART (http://smart.embl-heidelberg.de). Different domains are specific colours as shown on the blocks. P5CR, pyrroline-5-carboxylate reductase; CD, cytidine deaminase; LCR, low-complexity region; RRM, RNA recognition motif; RBD, RNA-binding domain. RBP11 and Tb927.10.15760 are negative controls, which did not have reproducible effects in the tethering screen.

Because of the possibility of false-positives (discussed below), it was important to validate some of our novel results using full-length clones. To do this, trypanosomes that constitutively expressed an mRNA encoding the CAT reporter with 5 *boxB* elements (*CAT-B-ACT*) were transfected with inducible lambda-N-myc fusion proteins. Most observed CAT activities were in qualitative agreement with the screening results (Figure 5). Quantitative agreement was not expected, since the measurement methods were so different. Cytidine deaminase was revealed as a false-positive, while ZC3H45 was a false-negative since tethering of full-length ZC3H45 decreased CAT expression. Remarkably, the full-length cap-binding protein eIF4E1 vastly decreased CAT expression, although it was negative in the PGKB experiment and a fragment had increased expression in the BLA screen (Supplementary Figure S4).

Concentrating on the novel proteins, the Tb927.7.2780 fusion increased reporter CAT activity around 7-fold, as observed for PABP1 [28]. This protein has short poly-glutamine and -histidine tracts (Figure 5A) and was previously found to be essential in a high-throughput RNAi screen [31]. It also co-purifies with two proteins previously shown to increase mRNA stability, ZC3H11 [10] and MKT1 [19]. Tethering of the full-length pyrroline-5-carboxylate reductase (P5CR, not in the list) caused a modest but reproducible 1.6-fold CAT activity increase (Figure 5). The hypothetical proteins Tb927.11.14220 and Tb927.11.8020 decreased reporter expression; interestingly, SCOP analysis detected an RNA-binding domain in Tb927.11.14220.

### Some of the novel regulatory proteins can bind RNA

Our screen had identified many proteins that affected mRNA translation or degradation when tethered artificially to an mRNA. In vivo, such proteins might be bound to mRNA directly, or via another protein. Alternatively, they might never be associated with mRNA, in which case the effect we had observed would be an artefact. To assess the RNA-binding properties of potential regulators, we constructed a custom protein array. We chose 384 proteins that are of interest in post-transcriptional regulation, including some translation factors, degradative enzymes, all proteins with identified RNA-binding domains and a subset of proteins that had been identified in the screen but had no known RNA-binding characteristics. For reasons of economy, we also excluded proteins of the mitochondrial inner membrane and matrix, cytoskeleton, vesicular transport pathway, surface proteins, nucleoporins, nuclear proteins, and a few proteins for which the ORFs were too large to be amplified by PCR. Gateway-compatible primers were designed (Supplementary Table S6), the ORFs were amplified, and the lengths of products confirmed by gel electrophoresis. The products were then re-amplified to create DNA templates for protein expression in a prokaryotic *in vitro* transcription-translation system. The resulting templates were spotted onto glass slides in triplicate, and then a transcription-translation mix was added to create protein arrays (Supplementary Figure S6). In order to minimise disruption of the protein structure our oligonucleotides included the native stop codon. This had the disadvantage that it was not possible to verify production of full-length protein by addition of a C-terminal tag. The templates did, however, encode an N-terminal His tag, production of which was verified (not shown).

The protein arrays were used for studies of RNA interactions by probing them with labelled total or poly(A)+ RNA (in each case two procyclic-form samples and one bloodstream-form sample). The results are summarized in Supplementary Table S7. Negative results cannot be interpreted since we did not test whether the full-length proteins were made and there is no way to know whether RNA-binding domains, if they exist, were correctly folded. A negative result would also be obtained if a protein has very sequence specificity and its target mRNAs have low abundance. For example, the PABPs and pumilio domain proteins were negative. As a positive control, we probed ZC3H11 with its cognate recognition sequence of (UAU) repeats; it showed a clear signal whereas a C→S mutant in the zinc finger failed to bind (not shown).

157 protein spots bound to total RNA in all three replicates, whereas 47 bound to poly(A)+ RNA (of which all but two were also positive on total RNA); 148 spots bound in at least 4 of the 6 experiments. The small RNA binding protein RBP3 gave the strongest signal (Supplementary Table S7); 43 other RNA-binding domain proteins were also positive in at least 5 slides. Proteins that showed some RNA binding included some with confirmed tethering activity (Figure 5) but no known RNA-binding domain: Tb927.10.15760 (5 slides), Tb927.1.3070 (5 slides), Tb927.7.2780 (total RNA only) and pyrroline-5-carboxylate reductase (3 slides). Other rather surprising positives were the two peroxins, PEX13 (3 slides) and PEX14 (5 slides), which are in the glycosomal membrane, and a variety of enzymes. From these results we concluded that many of the proteins that we had identified in our tethering screen - including those with no annotated RNA-binding domains - may indeed be capable of binding RNA. The remainder may influence mRNA or translation via interactions with other RNA-associated proteins.

## Discussion

In this study we have shown that genes regulating mRNA turnover and translation can be identified using a functional genomics approach. The screen identified mRNA regulation by canonical RBPs and by proteins that act via multi-protein complexes, and also revealed nearly 100 proteins that can bind RNA but had no previously known RNA-related function. The random shotgun approach has the additional power of allowing delineation of functional domains.

The tethering approach is very powerful but, as in all high-throughput screens, false-positives and -negatives can occur:

- The assay relies on the tethering of protein fragments to a reporter RNA. Most of the fragments come from proteins that have no way to interact with RNA *in vivo*, either directly or indirectly. The protein might be in the cytosol, but have no RNA-binding domains and no interactions with any other RNA-binding protein. Alternatively, it may normally be in another compartment. A positive score could arise from a chance interaction of this protein with some other component of the translation or degradation machineries. For example, mitochondrial proteins were found in both positive and negative categories. Proteins with nuclear targeting signals might retain the mRNA in the nucleus, inhibiting translation.
- The use of fragments can result in abnormal or defective protein folding. This may expose interacting domains that are normally concealed within the full-length, properly folded protein, giving a false-positive result. Alternatively, it may prevent normal function of the protein, giving a false-negative result. The fragments will also give a false-negative result if the entire protein is required for activity. A false-negative result is particularly likely if (i) an open reading frame of more than about 1.5 kb is required; (ii) the domain that is required for activation is very near the N-terminus; or (iii) the protein is very short, so few in-frame fusions are present. For example, full-length MKT1 strongly increases expression in the *CAT* tethering assay, but both the N- and C-terminal portions are required [19] and the open reading frame of over 3 kb is too long to be included in our library. Consequently, only a single clone showed any selection, in just one experiment; considering the whole open reading frame, no selection for MKT1 was apparent at all. The above disadvantages could be abrogated by using a library of full-length open reading frames: a resource that is already available for budding yeast [46] and humans [47]. N-terminal tagging may however abolish protein activity, for example if a native N-terminus is required for interactions.
- Scoring of the screen relies on PCR. Genes encoding repetitive proteins could give artifactually high scores since reads map more than once. Some cytoskeletal proteins that scored in our assays might fall into this category. Easily amplified sequences may also be artifactually enriched.
- A false-negative result will be obtained if the protein acts as part of a complex, but the other components are present in limiting amounts.

Despite these possible problems, the screen was overall highly informative. We envisage that this technique could be adapted to screen libraries for proteins that play a role in post-transcriptional control in other organisms where transfection methods with high efficiency are available. For *T. brucei*, this forward genetic approach could be easily used to identification of genes and pathways regulating other complex biological phenomena such as quorum sensing, antigenic variation, or drug target identification. Our set of 384 ORFs already provides a Gateway-compatible resource for proteins potentially involved in mRNA metabolism.

The RNA binding of yeast [48] and mammalian [49] proteins has previously been examined using microarrays, but it is not clear which of the identified RNA binders was also found in the datasets obtained by poly(A)+ mRNA precipitation. Protein microarrays have the advantage (unlike poly(A)+ mRNA precipitation) of being unaffected by *in vivo* protein abundance; however, false negatives may be caused by incorrect protein folding and there is the danger of un-physiological interactions such as electrostatic binding of basic proteins to nucleic acids. Nevertheless, the results do provide preliminary indications which could be followed up by *in vivo* RNA cross-linking studies. In future, probing with more specific sequences may allow us to determine the RNA-binding specificities of the candidate proteins.

The results described in this paper will be most useful in the context of other available datasets. A genome-wide RNAi screen provided a catalogue of *T. brucei* genes whose knock-down is detrimental to the parasite under a variety of developmental conditions [31]. This can be now be used, for example, to look for proteins that are essential in only one life-cycle stage, and are implicated in the post-transcriptional control of gene expression (These results are included in Supplementary Table S7). For bloodstream forms, these would include ZC3H5, ZC3H15, and numerous proteins of no known function. An RNAi screen for proteins associated with the AMP/cAMP response identified a number of proteins whose association with either signalling or control of gene expression were not apparent [50]. Of these, Tb927.11.2250 and Tb927.9.4080 are both up-regulators and Tb927.11.2250 binds RNA. Adenylosuccinate synthetase was suggested to affect differentiation via AMP metabolism: we also identified it as a weak expression down-regulator with RNA-binding properties.

The catalogues of mRNA-associated proteins that are already available for mammals [4], [5], [7] and yeast [6] include numerous metabolic enzymes. Application of our methods to Opisthokont systems would clearly facilitate interpretation of these mRNA-associated protein datasets. We have now documented potential regulatory functions for trypanosome cystathione gamma lyase (CTH), deoxyribose-phosphate aldolase, pyrroline-5-carboxylate reductase (P5CR), dihydrofolate reductase-thymidylate synthase (DHFR-TS), adenylosuccinate synthetase and tryparedoxin (TXN); all but the first two also bound RNA in the protein microarray assay. In Hela cells, two other oxidative stress-related enzymes - thioredoxin and peroxiredoxin - have been shown to bind RNA [4]. In animal cells, thymidylate synthase (TS) and dihydrofolate reductase (DHFR) bind to their own mRNAs, causing translational repression [51]. Here we showed that in trypanosomes, the bifunctional trypanosome DHFR-TS protein is also able to bind RNA and, as in animal cells, the functional consequence of its association with mRNA is inhibition of expression.

Results from yeast-two-hybrid interaction screens and affinity purifications will also assist with mechanism prediction. Proteins that showed activity in the screen but cannot themselves bind to RNA, may nevertheless be associated with RNA via interactions with RNA-binding proteins. For example, although the Tb927.9.4080 protein may not bind to RNA, it is known to interact with both DRBD3 [34] and MKT1 [19]. Down regulators might be expected to interact with components of the degradation machinery, or to have inactivating interactions with the translation apparatus. Proteins that enhance gene expression might be expected to be found stably associated with mRNA, and those that enhance translation ought to be at least partially associated with polysomes, so additional datasets of this sort will greatly facilitate interpretation of our data.

## Materials and Methods

### Plasmid construction and transgenic trypanosomes

The overexpression library was constructed in a derivative plasmid of the tetracycline-regulated pHD678 [52]. It contains a lambda-N peptide sequence cloned as a *Hind*III-*Apa*I fragment, and the *NEO* (neomycin/G418 resistance) gene replaced a *HYG* (hygromycin resistance). Specific oligonucleotides were ligated into the *Apa*I and *Bam*HI sites to add a stop codon in all three possible reading frames downstream of the unique *Xho*I cloning site. Reporter blasticidin plasmids are derivative of the pHD330 [53]. They contain a GFP gene positioned upstream of a blasticidin (*BLA*) resistance marker and were designed for targeting into the tubulin locus. For the screening, 5 copies of the *boxB* sequence element were inserted immediately between the reporter sequences and the actin 3′-UTR. A similar construct lacking the boxB element between the *BLA* and the 3′-UTR was used as control. For the PGKB experiment, the full-length PGKB CDS was cloned into the pHD2300. It confers *BLA* resistance and was designed for targeting into the *RRNA locus*. As described, 5 copies of the *boxB* element were embedded between the *PGKB* sequence and the actin 3′-UTR. Details of all plasmids and oligonucleotides are provided in Supplementary Table S6, sheet 2. Complete sequences are available from us upon request.

### Construction of the DNA expression library

The overexpression library was essentially made as previously described [19]. The plasmid was linearized at the unique *Xho*I site, filled in with the large fragment of DNA polymerase I in the presence of dTTP, and ligated to the semi-Xho-adapted DNA (size range from 0.7–3 Kbp). The ligation reaction was used to transform *Escherichia coli* NEB 5-alpha cells by electroporation to generate a library of approximately 3×10^6^ ampicillin-resistant colonies. To assess the quality of the library, *E. coli* cells were re-transformed with purified library, plated and plasmids purified from individual colonies. After *Xho*I digestion, inserts were found in 99% of plasmids and the average size was 1.2 Kbp.

### *T. brucei* growth and manipulation

Bloodstream-form *T. brucei* 2T1 cells were maintained and transfected as described [31] except that Tb-BSF buffer [54] was used for all transfections. The pRPaSce* plasmid was used to derive Sce* cells from 2T1 cells as described [31]. The Sce* strain expresses the tetracycline repressor (TetR), and inducible homing endonuclease (I-SceI) which facilitates site-specific overexpression library integration. Upon transfection, overexpression plasmid library constructs replace the I-SceI gene and cleavage site. Parasite libraries were generated by several rounds of electroporation and for each series, an aliquot of the transfection was diluted to determine the transfection efficiency. The average efficiency was about 5×10^−3^. To assay for stabilizing proteins, populations expressing the blasticidin resistance mRNA were pre-induced for 24 h in 1 µg/mL tetracycline and then grown with various concentrations of blasticidin (1x = 5 µg/mL) for four days.

### DNA sequencing and analysis

The PCR-amplified DNA was fragmented and sequenced using Illumina HiSeq with multiplexing and data analyzed as described previously [10], [55]. We sequenced input trypanosome population twice since the BLA-selected trypanosome libraries were expected to be less complex. SAMtools [56] and custom-made PERL scripts were used to select only coding region or 5′-UTR sequences that were in frame with the lambda-N peptide. The lambda-N sequence was removed, then the remainder was mapped to the *T. brucei* 927 reference genome (http://tritrypdb.org/tritrypdb) using Bowtie, allowing one base mismatch.

### Data analysis

To find proteins that increased blasticidin resistance we chose locations from position −36 relative to the ATG to position −18 relative to the stop codon (Supplementary Table S1). The counts ranged from 166000 to over a million (Supplementary Table S1). Then only positions for a unique gene set ([32], modified) were chosen. For these we counted, for each experiment, the reads per million (RPM). To avoid zero values, 1 was added to each value to give RPM+1 (Supplementary Table S2, sheet 2). After that, for each location, the number of counts for 6-fold increased blasticidin (BLA 6X) in experiment A was divided by the counts for −tet, and separately by the counts for cells with tetracycline but without blasticidin. To reduce the likelihood of identifying PCR artifacts, the lower of these two values was taken to be the relative enrichment for this site. Similar calculations were done for all locations for BLA10X in experiments B and C (Supplementary Table S2, sheet 3). Experiment C was the -boxB control, which should select only for fusions that enhance growth independent of the blasticidin resistance mRNA, and also for PCR artifacts. We therefore removed all locations that gave at least 3x increase in BLA10x in Experiment C. From the remaining list we selected genes for which at least two locations gave a 3x increase in either BLA6x (A) or BLA10x (B) (Supplementary Table S2, sheet 3). The number of such locations per ORF was counted (Supplementary Table S2, sheet 4).

Separately, and using a similar procedure, numbers of total counts for the whole coding regions were extracted (Supplementary Table S2, Sheet 5). For the unique genes, we computed the RPM in each experiment (Supplementary Table S2, Sheet 2). The RPM+1 with 6x or 10x blasticidin, relative to amounts without blasticidin were calculated as before (Supplementary Table S2, Sheet 3). We now deleted any CDS giving at least three-fold enrichment in counts for BLA10x in negative control experiment C. We retained CDSs that gave an overall count enrichment of at least 3-fold in both experiment A (BLA6x) and experiment B (BLA10x). The resulting 197 genes are listed in Supplementary Table S2, sheet S4 and (for readers who want a smaller and simpler file) in Supplementary Table S3, Sheet 1. Table 1 shows only the RBPs and translation factors that reproducibly increased BLA expression.

To find proteins that increased survival after PGKB expression, we took the counts for each location (Supplementary Table S4, sheet 1), computed the RPM and added 1 to every value to avoid zero values (Supplementary Table S4, sheet 2). We then divided the number of counts for +tet by the counts for −tet (Supplementary Table S4, sheet 3). The highest ratio for each location was found. Now, for each unique gene, we counted the number of locations that gave at least 3-fold enrichment, + tetracycline, in at least one experiment (Supplementary Table S4, sheet 3, column D). Separately, numbers of total counts for the whole coding regions were extracted (Supplementary Table S5, Sheet 1). For the unique genes, we computed the RPM in each experiment, added 1 as before, and ratios +tet to −tet were calculated for each experiment (Supplementary Table S5, Sheet 2). These were transferred to Supplementary Table S4, sheet 3 columns E–F. In addition, we looked for the effects of blasticidin selection. Counts per CDS with BLA1x (experiment A) or BLA2x (experiment B) were divided by the values with no blasticidin and the higher value taken. These values were also transferred to Supplementary Table S4, sheet 3, columns H, I and J. We now chose genes that were candidates as down-regulators. First, we selected genes for which at least two locations gave 3-fold enrichment in at least one of the PGK experiments (Supplementary Table S4, sheet 4, column D). From these, we selected genes that gave a 2-fold RPM increase over the whole coding sequence in at least two of the three PGKB experiments. This list of 127 genes is in Supplementary Table S4, sheet 4. The best regulators are those which gave selective advantage in the PGK experiment, and were selected against in the BLA experiment. Supplementary Table S3, sheet 2 has the same information, without the calculations and the raw data.

### Protein microarrays

Gene-specific primers were designed and synthesized in 96 well plates (Supplementary Table S6). The corresponding ORFs were then amplified directly from genomic DNA using Q5 high fidelity DNA polymerase (New England Biolabs), with 2 min at 98°C, 35 cycles (98°C for 15 sec, 52°C for 15 sec and 72°C for 2 min) then 10 min at 72°C. Each primer included additional sequence suitable for amplification and directional Gateway cloning. In a second PCR amplification, these extra sequences on the amplification products were used in order to reamplify the genes while adding a T7 promoter and Shine-Dalgarno sequence upstream of the ATG (Supplementary Table S6), with 0.02 µL of the first PCR as template (98°C for 2 min, two cycles of 98°C for 15 sec, 45°C for 15 sec and 72°C for 2 min; then 35 cycles as with the initial PCR). These templates were then used for the production of protein microarrays as previously described [57]. Briefly, 900 pL of DNA template was arrayed in triplicates on Nexterion Epoxy slide E using a GeSIM non-contact nanoplotter. In a subsequent spotting round, 3.6 nL of the S30T7 high yield protein expression system was spotted onto of each template spot. The entire assembly was placed in a deep humidified chamber containing 50 µL of nuclease-free water in each of its wells, and then incubated at 37°C for one hour and a 30°C overnight. Slides were removed from the chambers, cleaned and stored at −20°C until use. Poly(A)+ RNA was isolated from total RNA using Nucleotrap mRNA purification kit as recommended by the manufacturer. Total and poly(A)+ RNA were biotin-labeled using Pierce RNA 3′ End Biotinylation Kit following the manufacturer's instructions.

### On-chip protein-RNA interactions

Protein arrays were removed from the freezer and placed directly into a blocking solution containing Hepes-KOH pH 7.9, 10% glycerol, 40 U of RNaseout RNase inhibitor, 1% BSA, 100 µM ZnCl2, 1x Halt Protease and phosphatase cocktail inhibitor, 40 µg/mL heparin, 1 mM DTT 50 µg/mL *E. coli* tRNA, 50 mM glutamic acid potassium salt, 0.1% Triton X-100 and 8 mM Magnesium acetate. The slides were blocked for 1 hour at room temperature, and then washed in washing buffer (Blocking buffer without BSA). Slides were then incubated with labeled RNA in blocking buffer, containing 0.5 mg/mL *E. coli* tRNA overnight at 4°C. The slides were removed from the incubation chambers and washed 3x twenty minutes in washing buffer, and 3x five minutes in Nuclease-free water. For detection of total and poly(A)+ RNA, the arrays were probed with cy3-labelled extravidin at a dilution of 1∶100. The slides were air-dried in a ventilated oven at 30°C for one hour and scanned in a Tecan power scanner with 75% laser power and 500% PMT gain. Images were saved as TIFF files and later loaded into Genepix 6.0 for data extraction. The mean background signal for each spot was subtracted from the mean spot intensity, and the average intensity for all triplicates of each sample calculated. As controls, a mutant zinc finger protein (ZC3H11 C→S) which has been shown to have no RNA binding activity [10], as well as the PCR negative control and expression mix alone were used.

## Supporting Information

Figure S1 Selection for proteins that increase expression. (**A**) Cumulative growth of the population in all four conditions analyzed. Bloodstream-form *T. brucei* cells expressing the *BLA-B-ACT* reporter were transfected by electroporation and the pool cultures from several transfections were combined into one large library consisting of ∼1.8×10^6^ independent transformants (Library A). After 24 h tetracycline induction, cells were grown for four days in various concentrations of blasticidin as indicated. Cells were counted daily and cumulative growth curves for each condition were plotted on a logarithmic scale. (**B**) Plasmid-specific PCRs were carried out on genomic DNA isolated from survival cultures. Examination of PCRs revealed similar-looking smears of many products for all populations. WT: non-transfected cells. (**C**) Cumulative growth of the population in all five conditions analyzed. Cells carrying the *BLA-B-ACT* reporter were transfected as in (A) and the pool cultures were combined into one large library consisting of ∼3.3×10^6^ independent transformants (Library B). Blasticidin concentrations are indicated. (**D**) Cumulative growth of the population in all five conditions analyzed. Cells expressing the *BLA-ACT* reporter (lacking boxB) were transfected as in (A) and the pool cultures from several transfections were combined into one large library consisting of ∼3.1×10^6^ independent transformants (Library C; control library). Blasticidin concentrations are as in (B). (**E**) Plasmid-specific PCRs were carried out on genomic DNA from resistant cultures. WT: non-transfected cells. Molecular sizes are indicated on the right.
(EPS)

Figure S2 Selection for proteins that decrease expression. (**A**) Schematic of the overexpression library and the growth conditions analyzed. A cell line expressing tetracycline inducible PGKB followed by 5 boxB copies at the 3′-UTR is transfected with the trypanosome N-tagged library and then, cells are grown under non-inducing (coverage) and inducing (expression inhibitors) conditions. (**B**) Cumulative growth curves. Cells were counted daily and cumulative growth curves for each condition were plotted on a logarithmic scale. For this, three independent experiments containing 0.65×10^6^ (Library PGKB-A), 1.2×10^6^ (Library PGKB-B) and 4.8×10^6^ (Library PGKB-C) clones were performed. After transfection, parasites were grown for 5 days in the presence or absence of tetracycline and then, genomic DNA was isolated from survival populations. (**C**) Plasmid-specific PCRs were carried out on genomic DNA isolated from survival cultures. WT: non-transfected cells. Molecular sizes are indicated on the right.
(EPS)

Figure S3 Regions of characterized *trans*-acting factors required for enrichment upon blasticidin selection. (**A**) All reads aligned to the *ZC3H20* sequence for each BLA experiment, with scale to the left. The protein is shown schematically below. (**B**) To identify the protein fragments responsible for the enrichment, only reads that were in frame with the lambda-N peptide were considered. For each location, the fold enrichment was calculated by dividing the normalized RPM values from blasticidin 6x- and 10x-treated cells (average values; from position −36 from ATG to −18 from STOP) by the values obtained from the untreated cells (*green*) (1 was added to each RPM value to avoid dividing by 0). For the control cells lacking the *boxB* element, the fold enrichment was obtained under the same growth conditions and calculated as above (*red*). The horizontal dotted line shows the three-fold enrichment cut-off. The corresponding protein structures are depicted below. (**C**) Similar results for previously characterized factors: ZC3H11, ALBA1, PUF9, ZC3H20, ZFP3, DRBD3 (PTB1), DRBD4 (PTB2), RBP33 (CSBP2), RBP3 and RBP42.
(EPS)

Figure S4 Translation initiation factors tethered to the 3′-UTR can increase gene expression. For each location, the fold enrichment was calculated by dividing the normalized RPM values from blasticidin 6x- and 10x-treated cells (average values; from position −36 from ATG to −18 from STOP) by the values obtained from the untreated cells (*green*). For the control cells lacking the boxB element, the fold enrichment was obtained under the same growth conditions (*red*). The horizontal dotted line shows the three-fold enrichment cut-off. The corresponding protein structures are depicted below.
(EPS)

Figure S5 Proteins that decrease gene expression. (**A**) All reads aligned to the *4E-IP* sequence for each PGKB experiment, with scale to the left. (**B**) To identify the protein fragments responsible for the enrichment, only reads that were in frame with the lambda-N peptide were considered. For each location, the fold enrichment was calculated by dividing the normalized RPM values obtained from induced cells (tet +) by the values from the non-induced cells (tet −). The horizontal dotted line shows the three-fold enrichment cut-off. The corresponding protein structures are depicted below. (**C**) Similar results for known down-regulators: XRNA, RBP10 and DRBD12.
(EPS)

Figure S6 Proteome array procedure and results. (**A**) Cartoon of the procedure for a single spot. (**B**) Image of our array stained with Sypro Ruby (left) and hybridised with poly(A) mRNA (right).
(EPS)

Table S1 Selection of clones with blasticidin: reads per location. For each location in the genome, the number of reads is shown. Only in-frame reads are included. Experiments A and B were done in a cell line in which 5 boxB sequences were in the 3′-UTR. In experiment C (negative control) the BoxB sequences were absent. The location is relative to the start codon. Tet-: no tetracycline; Bla_0x: with tetracycline, without blasticidin. Other samples are grown with multiples of the normal (1x = 5 µg/mL) amount of Blasticidin for four days, before harvesting and amplification of the inserts.
(XLSX)

Table S2 Selection of clones with blasticidin: reads per open reading frame. Sheet 1: For each open reading frame, the total number of reads is shown (sum of reads from all in-frame locations in Supplementary Table S1). Experiments A and B were done in a cell line in which 5 boxB sequences were in the 3′-UTR. In experiment C (negative control) the BoxB sequences were absent. Tet-: no tetracycline; Bla_0x: with tetracycline, without blasticidin. Other samples are grown with multiples of the normal (1x = 5 µg/mL) amount of Blasticidin for four days, before harvesting and amplification of the inserts. Note that some sample libraries were sequenced twice to increase read depth. Sheet 2: Only unique genes (or a single copy of repeated genes) are included. Results are shown as reads per million reads (RPM). Sheet 3: Calculations. 1 was added to each RPM value to eliminate zero values. For each selection with BLA, there were two controls: −tet, and +tet. To find genes giving increased blasticidin resistance: The value for A_BLA_6x was divided by those for A_Tet- and A_BLA_0 (columns R and S) and the smaller value was chosen (column X). Similarly, the values for B_BLA_10x were divided by those for B_Tet- and B_BLA_0 (columns T and U) and the smaller value was chosen (column Y). As control, the values for C_BLA_10x were divided by those for C_Tet- and C_BLA_0 (columns V and W) and again the smaller value was chosen (column Z). To find locations with at least three-fold increase after selection, a similar procedure was used with Table S1. (These calculations have been deleted to reduce the file size.) The number of locations with such an increase was then counted. The result is in column AJ. To find genes giving increased blasticidin susceptibility: The value for A_BLA_1x was divided by those for A_Tet- and A_BLA_0 (columns AA and AB) and the larger value was chosen (column AG). Similarly, the values for B_BLA_2x were divided by those for B_Tet- and B_BLA_0 (columns AC and AD) and the larger value was chosen (column AH). As control, the values for C_BLA_2x were divided by those for C_Tet- and C_BLA_0 (columns AE and AF) and again the larger value was chosen (column AG). To find locations with at least two-fold decrease after selection, a similar procedure was used with Table S1. (These calculations have been deleted to reduce the file size.) The number of locations with such an increase was then counted. The result is in column AK. Sheet 4: List of genes in which at least two locations gave >3x increase in read counts, the average of BLA6x and 10x/control is at least 2, and each individually is >1. Sheet 54: List of genes in which at least three locations gave >3x increase in read counts, and BLA6x and 10x/control are both at least 3. The effect is also at least 2x higher in the presence of BoxB.
(XLSX)

Table S3 Summary of the best regulators. This Table summarizes the results from Supplementary Tables S2 and S5.
(XLSX)

Table S4 Selection of clones after induction of PGKB expression: reads per location. Sheet 1: For each location in the genome, the number of reads is shown. Only in-frame reads are included. Results for three experiments: A, B and C, are shown. The location is relative to the start codon. −tet: no tetracycline; +tet: with tetracycline Sheet 2: Only unique genes (or a single copy of repeated genes) are included. Results are shown as reads per million reads +1. Sheet 3: Calculations. The number of locations with a 3x increase (column D) is derived from sheet 2. Columns E–G show, for the individual experiments, the total reads per million reads (RPM) in the presence of tetracycline divided by the RPM without tetracycline (derived from Supplementary Table S5. Columns H–J show the results for BLA1x/control (BLA experiment A, Table S2, sheet 3) or BLA2x/control (BLA experiments B & C, Table S2, sheet 3). Column K is the number of PGK experiments in which the overall RPM increased at least 2-fold. Column L is the number of BLA experiments (A+B) in which the overall RPM increased at least 2-fold, minus 1 if the same happened in experiment C. Column M is the sum of K and L. The average RPM change for all three PGKB experiments is in column N, with the standard deviation in column O. The average change in the BLA experiments (A and B) in column P. Sheet 4: Proteins that decrease expression when tethered. Data are the same as in sheet 3, but filtered. The listed proteins have at least two locations with at least 3x increase +tet in the PGKB experiment, and an overall increase of at least 2x +tet in at least two PGKB experiments. Blue values are at least 2-fold increased (for PGKB) or decreased (for BLA). Red values are changed in the opposite direction.
(XLSX)

Table S5 Selection of clones after induction of PGKB expression: reads per open reading frame. Sheet 1 contains all reads. Sheet 2 - results for the unique gene set. Columns D–I are the raw reads, J,K,O,P And Y,U are the reads per million reads, and L,M,Q,R and V, W the RPM+1. The values for +tet/−tet are in columns N, S and X for experiments A, B and C respectively, and the average of all three is in column Y.
(XLSX)

Table S6 Oligonucleotides used to amplify open reading frames. Blue letters are Gateway-compatible tags for re-amplification. The start codon is in red.
(XLSX)

Table S7 Binding of RNA to a protein microarray. Sheet 1: Column B: mean signal for 3 replicate spots on the first slide incubated with procyclic poly(A)+ RNA. Column C: standard deviation for the 3 spots; Column D: P-value (probability that the signal is the same as the controls). Column E: signal divided by the control. Columns F–I and J–M are the same for the second procyclic replicate and for a bloodstream form sample. Column N: mean of all three ratios (columns E, I and M); Column O: number of slides positive. The criterion for a positive score was a p-value of less than 0.05, and a ratio of at least 1.5. Sheet 2: as for sheet 1, but using total RNA as probe. Sheet 3: Combined results for sheets 1 and 2. Column D: Results from the RNAi screen of Alsford *et al.* [31]; B3 - growth disadvantage in bloodstream forms after 3 days with tetracycline; B6 - growth disadvantage in bloodstream forms after 6 days with tetracycline; D - growth disadvantage in differentiating bloodstream forms with tetracycline; P - growth disadvantage in procyclic forms with tetracycline; b3 - growth advantage in bloodstream forms after 3 days with tetracycline; b6 - growth advantage in bloodstream forms after 6 days with tetracycline; d - growth advantage in differentiating bloodstream forms with tetracycline; p - growth advantage in procyclic forms with tetracycline. Column E: association with MKT1 [19]; Y2H = positive in the yeast 2-hybrid screen; TAP = positive by tandem affinity purification. Column F: average coding region read ratio in the PGKB screen; Column G: average coding region read ratio in the BLA screen; No slides positive = total number of proteome array slides giving a positive signal. Column I = column N on sheet 2; Column J = column N on sheet 1.
(XLSX)
